# Postoperative Dietary Intake Achievement: A Secondary Analysis of a Randomized Controlled Trial

**DOI:** 10.3390/nu14010222

**Published:** 2022-01-05

**Authors:** Chiou Yi Ho, Zuriati Ibrahim, Zalina Abu Zaid, Zulfitri Azuan Mat Daud, Nor Baizura Mohd Yusop, Mohd Norazam Mohd Abas, Jamil Omar

**Affiliations:** 1 Department of Dietetics, Faculty of Medicine and Health Sciences, Universiti Putra Malaysia, Seri Kembangan 43400, Malaysia; agneshcy0326@gmail.com (C.Y.H.); zalina@upm.edu.my (Z.A.Z.); zulfitri@upm.edu.my (Z.A.M.D.); baizura@upm.edu.my (N.B.M.Y.); 2 Department of Dietetics and Food Service, Institut Kanser Negara, Ministry of Health, 4, Jalan P7, Presint 7, Putrajaya 62250, Malaysia; 3 Department Surgical Oncology, Institut Kanser Negara, Ministry of Health, 4, Jalan P7, Presint 7, Putrajaya 62250, Malaysia; m_norazam@yahoo.com (M.N.M.A.); drjamil@nci.gov.my (J.O.)

**Keywords:** postoperative recovery, dietary intake, predictors

## Abstract

Sufficient postoperative dietary intake is crucial for ensuring a better surgical outcome. This study aimed to investigate the postoperative dietary intake achievement and predictors of postoperative dietary intake among gynecologic cancer patients. A total of 118 participants were included in this secondary analysis. Postoperative dietary data was pooled and re-classified into early postoperative dietary intake achievement (EDIA) (daily energy intake (DEI) ≥ 75% from the estimated energy requirement (EER)) and delay dietary intake achievement (DDIA) (DEI < 75% EER) There was a significant difference in postoperative changes in weight (*p* = 0.002), muscle mass (*p* = 0.018), and handgrip strength (*p* = 0.010) between the groups. Postoperative daily energy and protein intake in the EDIA was significantly greater than DDIA from operation day to discharged (*p* = 0.000 and *p* = 0.036). Four significant independent postoperative dietary intake predictors were found: preoperative whey protein-infused carbohydrate loading (*p* = 0.000), postoperative nausea vomiting (*p* = 0.001), age (*p* = 0.010), and time to tolerate clear fluid (*p* = 0.016). The multilinear regression model significantly predicted postoperative dietary intake, F (4, 116) = 68.013, *p* = 0.000, adj. R^2^ = 0.698. With the four predictors’ recognition, the integration of a more specific and comprehensive dietitian-led supportive care with individualized nutrition intervention ought to be considered to promote functional recovery.

## 1. Introduction

Postoperative catabolic reaction succeeding post-operation may weaken immune function, reduce muscle strength, prolong wound healing, and cause body skeletal muscle tissue catabolism. Surgery-related stress and inadequate postoperative dietary intake might cause extraneous fatigue and prolong convalescence [1]. Postoperative malnutrition is a worrisome shift in delaying recovery in cancer patients and might impact the rate of survival.

The postoperative nutritional requirement is higher to support anabolism and minimize nutritional depletion [2]. Adequate dietary intake is an important key point to achieve optimum nutritional status post operation to speed up the wound healing process, enhance immunity, and assure a better postoperative outcome [3]. The previous Randomized Control Trial (RCT) demonstrated that Enhanced Recovery After Surgery (ERAS) with preoperative whey protein-infused carbohydrate (CHO) loading and postoperative early oral feeding showed positive outcomes, nutrition status preservation, and suppressed inflammatory response without increasing postoperative complication [4]. The further investigation in this trial for subgroup effects regarding postoperative dietary intake was worth determining.

A prospective cross-sectional study investigated postoperative dietary intake from completion of the operation to discharge. Total daily energy (kcal) and protein (g) intake for each subject were analyzed and categorized as adequate if intake met ≥75% estimated requirements [5]. The study found 58.4% of patients started to have their postoperative first oral intake and first solid food intake on the operation day, respectively. About 53% of patients consumed clear liquids as the first drink after an operation of gastrointestinal patients [6]. The postoperative early dietary intake achievement (EDIA) could be promoted by introducing oral nutrition supplement (ONS). The postoperative enteral nutrition has been revealed to boost dietary intake, lessen morbidity, and reduce hospital stays [7]. As a result, the implementation of early postoperative enteral nutrition is conceivable to promote recovery and prevent body protein (muscle) catabolism. However, Henriksen et al. (2003) found that small, positive but not significant differences in body composition changes, dietary intake, and return of bowel function in preoperative CHO loading as compared to the fasting group [8].

To the best of our knowledge, there is limited previous study or RCT comprehensively examining the effects of dietitian-led supportive care on nutrition achievement and predictors of postoperative dietary intake in Malaysia. Progress in postoperative dietary intake has remained inconclusive. The current study therefore aimed to demonstrate the effects of dietitian-led supportive care on nutritional achievement and predictors of postoperative dietary intake.

## 2. Materials and Methods

### 2.1. Study Design

This is a secondary analysis of the previous RCT on the impact of ERAS with whey protein-infused carbohydrate loading and postoperative early oral feeding vs. standard care among GC patients admitted for an elective operation, which was undertaken from November 2017 to September 2019 [9]. The secondary analysis was conducted from January 2020 to March 2020. All data was pooled, re-classified and focused on the postoperative nutritional achievement and predictors of postoperative dietary intake among GC patients. The rationale for the RCT, design details, and eligibility characteristics, as well as the primary results, have been published previously [4].

### 2.2. Participants

**Figure 1:**
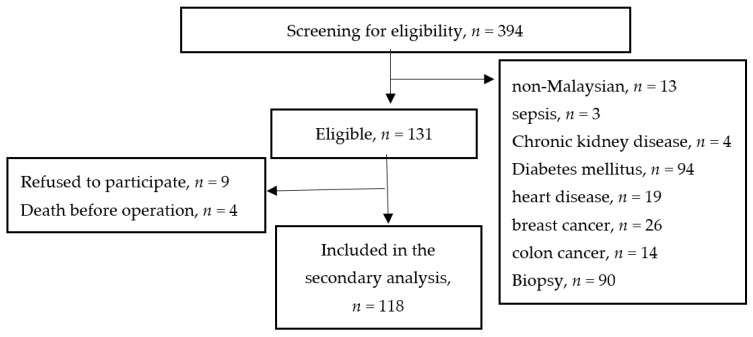
Flow Diagram of the Subjects’ Selection Process and Specific Reasons for Exclusion.

This secondary analysis included all 118 consenting participants who were recruited in the RCT (Figure 1). The inclusion criteria for RCT were ambulated Malaysian aged 18 years and above scheduled for elective surgery for suspected GC, while exclusion criteria were physical disability, soy or whey protein allergy, diagnosed with chronic kidney disease, ischemic heart disease, diabetes mellitus, or involved in other intervention studies.

### 2.3. Outcomes Measurement

#### Participant Group and Study Endpoint

For the postoperative period, the participants’ energy requirements were calculated based on the recommended formula to estimate energy requirements for cancer patients [3]. Postoperative daily dietary intake ≥ 75% EER is considered adequate [5], essential to prevent further nutrition depletion, and promote wound healing [10] and reduced infection risk [11]. Hence, we pooled and re-classified participants by using the distribution of total daily energy intake per estimated energy requirement (EER) on postoperative day-two. In this secondary analysis, participants were defined into two groups which were early dietary intake achievement (EDIA) (daily energy intake ≥ 75% EER) and delay dietary intake achievement (DDIA) (daily energy intake < 75% EER). The primary endpoint of the study was postoperative nutritional achievement and predictors of the postoperative dietary intake on postoperative day-two.

Percentage of dietary intake achievement (%) = total daily energy intake/estimated energy requirement

### 2.4. Sociodemographic and Clinical Characteristics

Sociodemographic (age) and clinical characteristics included primary diagnosis, cancer stage, comorbidities, family history of cancer and American Society of Anesthesiologists (ASA) score, tracked and recorded from the electronic medical record system.

### 2.5. Nutritional and Functional Status

Anthropometric and functional status (handgrip strength) measurements were assessed during admission and upon discharge. Body weight was measured according to the procedures described [12]. Weight, fat percentage, fat mass, fat-free mass, and muscle mass of subjects were assessed by the body composition analyzer TANITA^®^ SC 300 (TANITA Corporation, Hoofddorp, The Netherlands). Height was measured with subject in standing position [12] with a scheduled calibrated SECA^®^ 769 (Seca GmBH & Co., KG, Hamburg, Germany). Body mass index (BMI) is defined as weight in kilograms (kg) divided by height in meters squared (m^2^). The calibrated digital JAMAR^®^ Handgrip Dynamometer (Asimow Engineering Co., Los Angeles, CA, USA) was used to assess handgrip strength. The average score of the three trials was used to interpret handgrip strength performance. These data were traced from the RCT database.

### 2.6. Biochemical Profile (Serum albumin)

Medical personnel (medical officer or staff nurse) drew the subject’s blood for investigation in the female surgical ward. Blood sample analysis was performed by the medical laboratory technologist at the Pathology Department, National Cancer Institute, Malaysia. The preoperative and postoperative serum albumin were traced from the RCT database.

### 2.7. Pre-Admission and Postoperative Dietary Intake

Preadmission and postoperative dietary intake, which was assessed using a 24-h diet recall method via a face-to-face interview by the dietitian, were traced from the RCT database. The 24-h diet recall was collected during admission and the postoperative days until discharge (daily in the ward). Food intake chart (food, beverage, or ONS if prescribed by a dietitian) was recorded by participants or staff nurses in charge in the ward. To verify dietary intake in the ward, the dietitian in charge (researcher) counterchecked the compliance (frequency and dilution) of ONS and amount of diet consumption during mealtime on the ward. Atlas of Food Exchanges and Portion Size [13], household measurements such as cups, spoons, and scoops and food models were used to assist participants in assessing the portion size of the foods they ate. Recorded dietary intakes were analyzed by using Nutritionist Pro Dietary Software version 2.4 (San Bruno, CA, USA) [14]. Energy intake in kilocalories (kcal) and protein intake in grams (g) were obtained from a summary of the analysis.

### 2.8. Postoperative Outcomes

The preoperative whey protein-infused CHO loading execution was tracked from the RCT database. The postoperative surgical outcomes included the method of operation, ICU admission, postoperative infection, postoperative nausea and vomiting (PONV), time to tolerate clear fluid, food toleration, and duration of hospital stay. The time to tolerate clear fluid was defined as the time from operation end to the time the patient could tolerate clear fluid. The time to tolerate food was defined as the time from operation end to the time the patient could tolerate solid food. The duration of hospital stays was defined as the time from admission to discharge. Postoperative outcomes were traced and recorded on a data collection form by a researcher.

### 2.9. Ethical Approval

In the previous RCT, eligible patients were provided with patient information sheet, study consent form and ample time to consider and discuss the participation with family members before decision making. The study was registered in the National Medical Research Registry Malaysia and Clinical Trial Registration with registration number NCT03667755 for publication purposes. The ethical approval of the study was received from the Medical Research Ethics Committee (MREC) with reference number NMRR-17-1070-36021.

### 2.10. Statistical Analysis

The analyses were conducted using IBM SPSS (version 23.0). Descriptive statistics were used for participants’ descriptive characteristics. Kolmogorov-Smirnov test and visual inspection of the stem-and-leaf plot confirmed that all variables were normally distributed. The Levene’s statistics were non-significant and thus the assumption of homogeneity of variances were not violated. The homoscedasticity was assessed and found to be supported. A visual inspection of normal Q-Q and detrended Q-Q plots for each variable confirmed that all were normally distributed. Therefore, the numerical data were presented as mean ± standard deviation while categorical data were presented as frequency and percentage. Since data were normally distributed, an independent t-test was used to compare the numerical variable between the groups. Pearson’s Chi-square test (with α = 0.05) was used to evaluate categorical data. The two-way mixed-model ANOVA was used to analyze the trend of postoperative dietary intake achievement between the groups. Pearson correlation coefficient was calculated where indicated. Significant univariate variables (*p* < 0.05) were entered into the multilinear regression analysis model to identify predictors of postoperative dietary intake achievement on postoperative day-two. All probability values were two-sided and a level of significance of less than 0.05 (*p* < 0.05) was considered statistically significant.

## 3. Results

**Figure 2:**
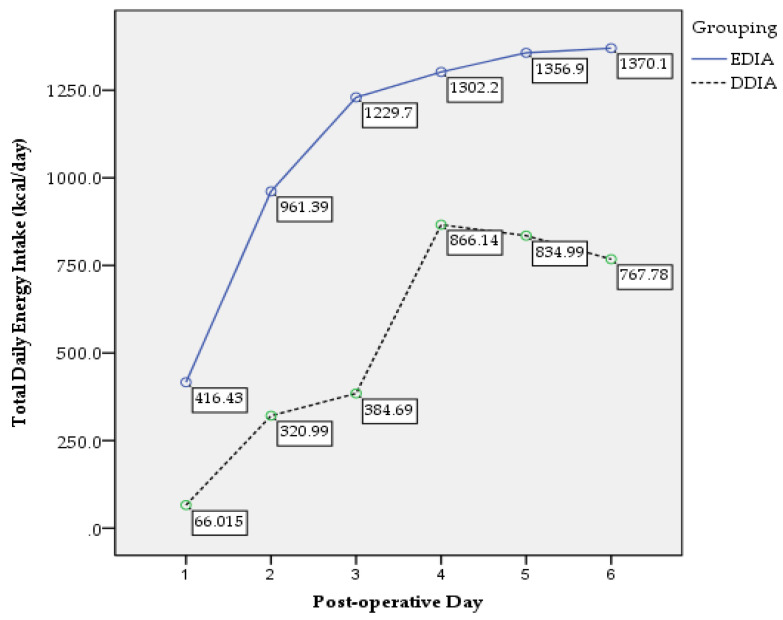
Postoperative total daily energy intake trend.

**Figure 3:**
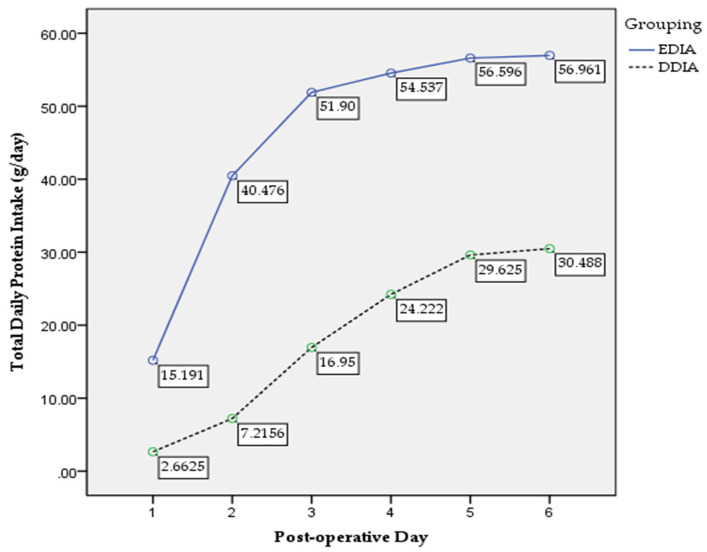
Postoperative total daily protein intake trend.

There were 46 (39%) and 72 (61%) participants in the EDIA and DDIA group, respectively. Means of age were 47.5 ± 11.9 years old for EDIA and 52.1 ± 11.8 years old for DDIA group. For clinical characteristics, nutritional and functional status, see Table 1. Table 2 demonstrates the postoperative surgical, nutritional and functional outcomes. There was significant difference in changes of weight (*p* = 0.002), muscle mass (*p* = 0.018) and handgrip strength (*p* = 0.010) between the groups. Figure 2 shows the trend in postoperative total daily energy intake between the groups. A significant main effect for group was found, F (1, 110) = 136.18, *p* = 0.000, partial eta squared = 0.558 with confidence level EDIA being significantly higher than DDIA. A significant interaction between time and groups was reported, F (2.82, 255.95) = 22.40, *p* = 0.000, partial eta squared = 0.172. Figure 3 presents the trend of postoperative total daily protein intake between the groups. A significant main effect for group was found, F (1, 111) = 204.67, *p* = 0.000, partial eta squared = 0.655 with confidence level EDIA being significantly higher than DDIA. A significant interaction between time and groups was reported, F (2.03, 244.54) = 1.56, *p* = 0.036, partial eta squared = 0.117.

Table 3 presents multivariate analysis analyzed all the significant parameters in the univariate analysis and revealed that four variables were statistically significant in contributing to the prediction. Hence, the significant independent predictors of postoperative dietary intake on postoperative day-two included preoperative whey protein-infused CHO loading (*p* = 0.000), PONV (*p* = 0.001), age (*p* = 0.010), and time to tolerate clear fluid (*p* = 0.016). The multilinear regression model statistical significantly predicted postoperative dietary intake achievement on postoperative day-two, F (4, 116) = 68.013, *p* = 0.000, adj. R^2^ = 0.698.

## 4. Discussion

Upon admission, more subjects in the current study were classified as overweight or obese based solely on BMI assessment. BMI and weight were unable to detect malnutrition among GC patients when used as the sole nutritional variable. This finding was consistent with previous studies in which BMI alone was limited in reflecting nutritional status and thus not accurate in indicating malnutrition in GC patients [15,16,17,18]. Underestimation of BMI in detecting malnutrition might be caused by body fluid imbalance, which includes the presence of ascites or oedema caused by a decrease in albumin [15,19].

The present study found that EDIA not only achieved higher and faster total daily energy and protein intake significantly throughout the hospitalization period but also experienced less weight and muscle depletion compared to DDIA. The prolonged preoperative fasting period was diminished by preoperative CHO loading with a whey protein-infused CHO drink and postoperative early oral feeding thus changed the body from a ‘fast’ state to a ‘fed’ state [20]. EDIA initiated earlier postoperative oral feeding as per ERAS recommendation. The majority of EDIA received intensive nutritional intervention where they were not only received the energy-dense clear fluid ONS preoperatively at 3-h, but also energy-dense clear fluid ONS postoperatively once they had started clear fluid and followed by energy-dense complete ONS after allowing solid diet. while most of DDIA received plain water after allowing for clear fluid and nourishing fluid, and followed by soft diet [21]. Postoperative intensive nutritional intervention management aimed to prevent nutritional depletion due to negative energy protein balance, and maintain an appropriate nutritional status to support rehabilitation and wound healing [3]. The integration of energy- and protein-dense ONS into the postoperative nutritional intervention regime intended to secure protein and energy intake while the oral intake was building [2,22].

The current finding was similar to the results from studies by Yeung et al. and Brown et al. where adequate energy and protein intake during the perioperative period prevent nutritional depletion and promote a speedy recovery [22]. Postoperative suboptimal energy protein intake increases the nutritional depletion rate if no further nutritional intervention is carried out [3]. Therefore, the free unrestricted diet was recommended from 4 h post-operation and ONS should be provided to ensure adequate postoperative energy and protein intake [23]. The postoperative patients, who rapidly progressed to standard diet immediately after 500 mL clear fluid toleration, achieved higher energy protein intake compared with those under slow progression conventional transition diet, with no significant increase in complication rate [24]. This indicated postoperative early oral feeding and rapid progression to a normal diet after tolerating clear fluid hastened diet toleration time, boosted postoperative total daily energy and protein intake, and cut down the reliance on ONS [25]. In addition, ICU admission showed negative effects, and delayed postoperative dietary intake might lead to clinically hemodynamic instability in initiating feeding and experiencing dysphagia after extubation [26]. The present study also demonstrated that the operation time influences the postoperative dietary achievement. Longer duration is usually associated with more complex operations, higher complications and prolonged recovery, as well as delayed postoperative dietary intake [27].

Dietary energy and protein intake were correlated with body composition including muscle mass. Inadequate oral intake might extend the catabolic response and further deplete the nutritional status post-operation [11]. Prolonged inadequate oral intake and hypercatabolic trigger skeletal muscle degradation [28]. Therefore, postoperative total daily energy and protein intake determine protein metabolism and muscle wasting. These approaches minimize the energy protein negative balance, provide early energy protein supply, reduce protein loss, improve muscle function, and promote the anabolic state. Preservation of postoperative weight loss and muscle wasting could be achieved from minimizing body glycogen breakdown, glucose synthesis from protein or fat, and fat oxidation [21]. Body composition and handgrip strength conservation might result from the combined effect of shortening of preoperative fasting and postoperative early feeding. Henrikson et al. also concluded that patients with preoperative CHO plus protein loading acquired greater muscle strength [8]. Beattie et al. identified a greater extent of muscle function preservation approaching those close to preoperative levels with early oral feeding with ONS [29].

In the present finding, there are four statistically significant independent predictive factors related to postoperative dietary intake achievement on postoperative day-two among surgical GC patients: age, pre-operative whey protein-infused CHO loading, presence of postoperative nausea and vomiting, and time to start clear fluid. Age influences post-operation dietary intake and tolerance. Old age was demonstrated as a risk factor of postoperative severe malnutrition [30]. Another study also revealed that old age and female patients were significantly associated with delayed postoperative oral toleration. This finding might be due to old female patients being more perceptive to gastrointestinal discomfort while initiating oral intake post-operation. They favor resuming and increasing oral intake gradually as compared to male or younger patients [31]. A study showed that geriatric patients experienced a higher risk of being malnourished post-operation [30]. Hence, postoperative intensive nutrition management by providing ONS was suggested among geriatric surgical patients to increase dietary energy and protein intake, prevent further nutritional depletion and shorten the duration of hospital stays [3].

PONV is a common reason for delayed functional recovery [32]. Anorexia or loss of appetite is a common reason for postoperative inadequate dietary intake related to gastrointestinal dysfunction and postoperative pain. Severe PONV, salivary secretion reduction, and change in taste could be induced by intubation, anesthesia, and surgery-related inflammation after the abdominal major surgery [33]. PONV was shown to be reduced with preoperative CHO loading [34]. An ERAS study in gynecological oncology showed that postoperative early oral feeding lessened abdominal distension, postoperative nausea, and vomiting and hastened gastrointestinal recovery [35]. The fear of PONV and specific food preferences might cause self-delay in postoperative feeding [6]. The patient-centered dietary approach, which included anti-emetics and prescription of unrestricted diet, may have assisted in commencing feeding. Early nutritional assessment to detect insufficient dietary intake, and intensive nutrition intervention to optimize dietary intake were recommended postoperatively. Intensive and individualized postoperative nutrition intervention improves dietary intake, enhances functional recovery, and prevents further nutritional depletion [36].

The present finding demonstrated that preoperative whey protein-infused CHO loading and starting clear fluids boosted the postoperative dietary intake. As per evidenced based ERAS recommendation, preoperative CHO loading lessened PONV and improved postoperative oral toleration [37,38]. Postoperative early oral feeding, which initiated clear fluid ingestion 4–6 h post-operation as one of the element of ERAS recommendation [39], stimulates early dietary intake and toleration by virtue of accelerating intestinal function recovery and prevents the occurrence of peristalsis of the stomach and small intestine and irregular contraction waves resulting from prolonged fasting. Thus, the intestinal mucosal barrier function could be maintained, further accelerating organ recovery [21]. Preoperative CHO loading was shown as a positive impact in minimizing insulin resistance and catabolism of muscle mass and subsequently resulted in the minimization of postoperative complications and preservation of nutritional status and muscle strength [37]. Yamada et al. also reported that preoperative CHO loading ensured better body weight preservation [40].

Current results did not show a correlation between preoperative nutritional status and postoperative dietary intake achievement. The perioperative nutrition approaches in the ERAS protocol (preoperative CHO loading and postoperative early oral feeding), PONV management, and age, all showed a greater impact on the postoperative dietary intake than preoperative malnutrition. Hence, the role of dietitian-led nutritional intervention after a major operation has been demonstrated to improve energy and protein intake. Perioperative dietitian-led nutritional management is crucial to optimize nutritional status [41]. Other than nutritional intervention management post-operation, individualized intensive nutritional intervention management with integration of ONS upon discharge is an essential element to be provided and explained to the patients and caregiver in order to achieve energy protein requirements and promote postoperative recovery [42].

### Strength and Limitations

The present study was the first to demonstrate postoperative nutritional achievement as well as investigate the predictors of postoperative dietary intake achievement among GC patients in Malaysia. However, the current study did have a few limitations. This was a single-center study observation that focused on surgical GC patients who underwent elective operation only. Thus, the predictive model may not suit other surgical cancer patients. The model might become superior if there were various types of cancer patient and multi-center involvement. The current study did not include other known factors from the literature in the multilinear regression model, such as surgical approach and cancer stage. Last but not least, selection bias might have occurred due to the fact that the operation may not have been offered to those with non-operable GC and severely malnourished patients.

## 5. Conclusions

Postoperative early dietary intake achievement not only assures a shorter duration of hospital stay but also preserves body composition among GC patients. The identification of postoperative dietary intake predictors stimulates the development of better multidisciplinary patient-centered ERAS approaches, incorporated into a more specific and comprehensive dietitian-led individualized intensive nutrition intervention management pre- and post-operation to promote postoperative functional recovery. This further suggests the crucial need for perioperative nutritional management among surgical cancer patients.

## Figures and Tables

**Table 1:** Clinical characteristics, nutritional status and function status in Gynecologic Cancer patients (*N* = 118).

Parameters	EDIA (*n* = 46)	DDIA (*n* = 72)	*p*-Value
Age (years) (mean ± SD)	47.5 ± 11.9	52.1 ± 11.8	^b^ 0.039 *
Primary diagnosis (*n*, %)			
Ovarian cancer	18 (39)	32 (44)	
Endometrial cancer	18 (39)	22 (31)	
Cervical cancer	8 (17)	13 (18)	
Uterine cancer	2 (5)	5 (7)	
Stage of cancer (*n*, %)			
1	42 (91)	64 (89)	
2	1 (2)	2 (3)	
3	1 (2)	0 (0)	
Advanced	2(4)	6 (8)	
Comorbidities (*n*, %)			^a^ 0.021 *
Hypertension	13 (28)	35 (49)	
Hypertension and dyslipidemia	1 (2)	12 (17)	
None	32 (70)	25 (34)	
ASA classification score (*n*, %)			^a^ 0.034 *
1	26 (57)	16 (22)	
2 & 3	20 (43)	56 (78)	
Preoperative nutritional status (mean ± SD)			
Weight (kg)	63.7 ± 12.7	65.9 ± 16.4	^b^ 0.419
BMI (kg/m^2^)	35.6 ± 6.1	37.1 ± 6.2	^b^ 0.193
Muscle mass (kg)	37.1 ± 4.0	37.3 ± 4.7	^b^ 0.808
Percentage weight loss within 1-month (%)	−3.3 ± 5.8	−5.9 ± 7.4	^b^ 0.041 *
Total daily energy intake (kcal/day)	1490 ± 247	1319 ± 355	^b^ 0.005 **
Total daily protein intake (g/day)	61.9 ± 15.8	53.3 ± 16.4	^b^ 0.006 **
Serum albumin level (g/L)	39.4 ± 4.4	37.4 ± 6.5	^b^ 0.053
Functional status (mean ± SD)			
Handgrip strength	17.0 ± 6.3	15.2 ± 6.0	^b^ 0.121

EDIA: Early Dietary Intake Achievement; DDIA: Delayed Dietary Intake Achievement; ASA: American Society of Anesthesiologists BMI: body mass index; PG-SGA: Patient-generated scored global assessment; ^a^ Chi-square test; ^b^ Independent *t*-test; * *p* < 0.05; ** *p* < 0.01.

**Table 2:** Postoperative surgical, nutritional and functional outcomes (*N* = 118).

Parameters	EDIA (*n* = 46)	DDIA (*n* = 72)	*p*-Value
Surgical outcomes			
Preoperative whey protein CHO loading (*n*, %)			^a^ <0.001 **
Yes	45 (98)	17 (24)	
No	1 (2)	55 (76)	
Method of operation (*n*, %)			^a^ 0.072
Laparoscopic	27 (59)	54 (75)	
Laparotomy	19 (41)	18 (25)	
ICU admission (*n*, %)			^a^ 0.001 **
Yes	1 (2)	17 (24)	
No	45 (98)	55 (76)	
Postoperative nausea and vomiting (*n*, %)			^a^ <0.001 **
Yes	7 (15)	53 (74)	
No	39 (85)	19 (26)	
Postoperative infection (*n*, %)			0.402
Yes	1 (2)	5 (7)	
No	45 (98)	67 (93)	
Operation time (mean ± SD)	2.3 ± 1.1	2.7 ± 1.2	0.031 *
Postoperative serum albumin (g/L) (mean ± SD)	32.5 ± 6.1	28.5 ± 6.0	^b^ <0.001 **
Time to start clear fluid (hours) (mean ± SD)	9.7 ± 2.9	19.7 ± 9.0	^b^ <0.001 **
Time to tolerate solid diet (hours) (mean ± SD)	21.3 ± 11.6	46.6 ± 19.6	^b^ <0.001**
Duration of hospital stays (hours) (mean ± SD)	114.6 ± 38.4	150.0 ± 30.1	^b^ <0.001 **
Nutritional outcomes			
Weight (kg)	−0.3 ± 2.5	−1.7 ± 2.3	^b^ 0.002 **
Muscle mass (kg)	0.4 ± 1.8	−0.5 ± 2.4	^b^ 0.018 *
Functional outcomes			
Handgrip strength (kg)	0.7 ± 4.0	−1.4 ± 4.8	^b^ 0.010 *

EDIA: Early Dietary Intake Achievement; DDIA: Delayed Dietary Intake Achievement; CHO: carbohydrate; ICU: Intensive care unit; ^a^ Chi-square test; ^b^ Independent *t*-test; * *p* < 0.05; ** *p* < 0.01.

**Table 3:** Predictors of postoperative dietary intake on postoperative day-two (*N* = 118).

Postoperative Dietary Intake on Postoperative Day-Two Summary Measure	Beta	95% CI	*p*-Value
Preoperative whey protein-CHO loading	0.552	407.532–693.712	<0.001 **
PONV	−0.210	−330.754–−87.173	0.001 **
Age	−0.127	−9.506–−0.993	0.010 **
Time to start clear fluid	−0.182	−18.347–−2.533	0.016 *

CHO: carbohydrate; PONV: postoperative nausea and vomiting; R = 0.842; R^2^ = 0.708, adjusted R^2^ = 0.698; F = 68.013, *p* = 0.000; Stepwise multilinear regression; * *p* < 0.05; ** *p* < 0.01.

## Data Availability

Data are available upon request.
